# Signal Transduction Mechanisms Underlying Group I mGluR-mediated Increase in Frequency and Amplitude of Spontaneous EPSCs in the Spinal Trigeminal Subnucleus Oralis of the Rat

**DOI:** 10.1186/1744-8069-5-50

**Published:** 2009-09-02

**Authors:** Ji-Hyeon Song, Eun-Sung Park, Sang-Mi Han, Seung-Ro Han, Dong-Kuk Ahn, Dong-Ho Youn

**Affiliations:** 1 Department of Oral Physiology, School of Dentistry and Brain Korea 21, Brain Science and Engineering Institute, Kyungpook National University, 188-1 Samduk-2-ga, Chung-gu, Daegu 700-412, Korea

## Abstract

Group I mGluRs (mGluR1 and 5) pre- and/or postsynaptically regulate synaptic transmission at glutamatergic synapses. By recording spontaneous EPSCs (sEPSCs) in the spinal trigeminal subnucleus oralis (Vo), we here investigated the regulation of glutamatergic transmission through the activation of group I mGluRs. Bath-applied DHPG (10 μM/5 min), activating the group I mGluRs, increased sEPSCs both in frequency and amplitude; particularly, the increased amplitude was long-lasting. The DHPG-induced increases of sEPSC frequency and amplitude were not NMDA receptor-dependent. The DHPG-induced increase in the frequency of sEPSCs, the presynaptic effect being further confirmed by the DHPG effect on paired-pulse ratio of trigeminal tract-evoked EPSCs, an index of presynaptic modulation, was significantly but partially reduced by blockades of voltage-dependent sodium channel, mGluR1 or mGluR5. Interestingly, PKC inhibition markedly enhanced the DHPG-induced increase of sEPSC frequency, which was mainly accomplished through mGluR1, indicating an inhibitory role of PKC. In contrast, the DHPG-induced increase of sEPSC amplitude was not affected by mGluR1 or mGluR5 antagonists although the long-lasting property of the increase was disappeared; however, the increase was completely inhibited by blocking both mGluR1 and mGluR5. Further study of signal transduction mechanisms revealed that PLC and CaMKII mediated the increases of sEPSC in both frequency and amplitude by DHPG, while IP_3 _receptor, NO and ERK only that of amplitude during DHPG application. Altogether, these results indicate that the activation of group I mGluRs and their signal transduction pathways differentially regulate glutamate release and synaptic responses in Vo, thereby contributing to the processing of somatosensory signals from orofacial region.

## Background

Orofacial somatosensory signals are transmitted to the first central relay site within the trigeminal sensory nuclear complex of brainstem via afferent component of the trigeminal nerve. The trigeminal complex consists of the principal nucleus and the spinal trigeminal nucleus. The latter is subdivided into oralis (Vo), interpolaris (Vi) and caudalis (Vc) in a rostro-caudal direction. The Vo receives somatosensory inputs from various oral and perioral structures including tooth [1,2], periodontal tissue[3], lip [4] and skin [5,6]. Recently, it has been suggested that the Vo is implicated in the central processing of nociceptive information owing to containing convergent and nociceptive-specific neurons, the neurons changing their properties by intramuscular mustard oil [5] and subcutaneous formalin [7]. Moreover, some Vo neurons transmit the processed somatosensory signals into other nociception-related brainstem areas, such as the parabrachial nucleus [8] and the thalamus [9,10]. Therefore, Vo is an important functional brainstem area participating in the processing and transmission of nociceptive information, resembling the deep laminae of the Vc and the spinal cord dorsal horn [6,8].

In the central nervous system (CNS), glutamate mediates fast excitatory synaptic transmission or exerts slow synaptic effects by binding its ionotropic (iGluR) or metabotropic (mGluR) glutamate receptors [11]. The mGluRs are one of G-protein coupled receptor families, enclosing eight subtypes (mGluR1-8) that can be divided into three groups according to sequence homology, pharmacology and signaling mechanisms [12].

Group I mGluRs (mGluR1 and 5) activate phospholipase C (PLC) via Gq-protein, resulting in phosphoinositide hydrolysis, Ca^2+ ^release from inositol 1,4,5-triphosphate (IP_3_)-sensitive intracellular stores, and protein kinase C (PKC) activation by diacylglycerol (DAG) [12]. On the contrary, group II (mGluR2 and 3) and III (mGluR4, 6-8) mGluRs are negatively coupled to cAMP production pathway via Gi/Go-protein [12]. Group I mGluRs are expressed in perisynaptic region of postsynaptic dendrites, and can mediate slow excitatory synaptic transmission in the CNS, including the spinal cord [13,14]. A previous study showed the expression of mGluR1 and mGluR5 in the trigeminal system including the subnucleus Vo [15]. mGluR1 was immunostained in the neuropil of all the trigeminal nuclei, and mGluR5-immunoreactive neurons are distinguishable in Vo. To date, although the precise synaptic localization and synaptic function of mGluR1 and 5 have been widely studied in various brain regions, including the spinal cord dorsal horn [16-21], the functions of group I mGluRs in the regulation of glutamatergic synaptic transmission in the Vo [15] have not been studied yet. Hence, we here attempted to study the functional roles of group I mGluRs in regulating glutamate release and modulating an α-amino-3-hydroxy-5-methylisoxazole-4-propionic acid (AMPA) receptor-mediated synaptic responses by recording spontaneous excitatory postsynaptic currents (sEPSCs) and trigeminal tract (Vt) stimulation-evoked EPSCs from the Vo neurons in the horizontal brainstem slices. In addition, we examined signaling pathways responsible for the group I mGluRs-mediated synaptic regulation.

## Results

### Increases of sEPSC frequency and amplitude and change of trigeminal tract-evoked EPSC amplitude by DHPG in Vo

**Figure 1:**
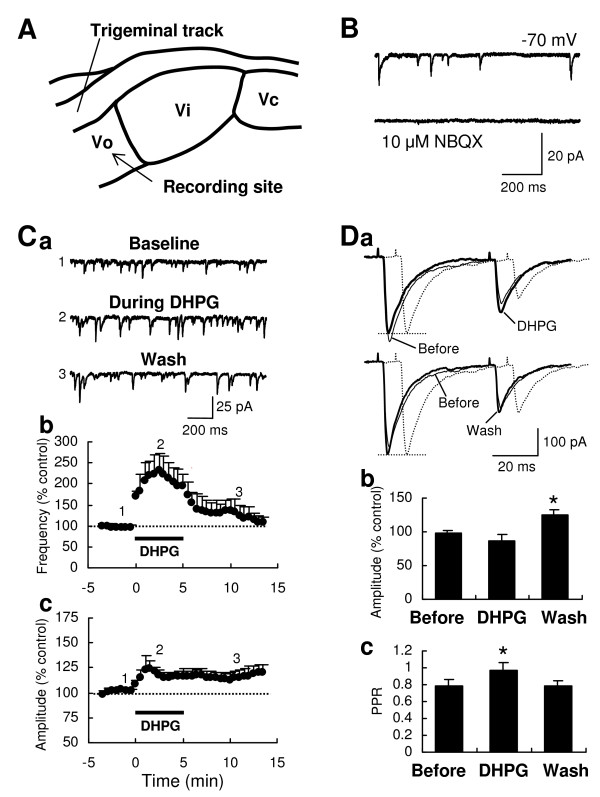
**Increase of sEPSC frequency and amplitude and change of Vt stimulation-evoked EPSC amplitude by bath application of DHPG in Vo**. A diagram (A) demonstrates the recording site of the Vo area within the spinal trigeminal nucleus and the electrical stimulation site in the Vt. sEPSCs recorded at holding potential of -70 mV in the Vo area were blocked by 10 μM NBQX (B). Representative sEPSC traces at -70 mV, recorded from Vo neurons with Cs-based internal solution, before and during DHPG (10 μM, 5 min), the group I mGluR agonist, and after washout of the drug (Ca). Time-course graphs demonstrate DHPG-induced increases of sEPSC frequency (Cb) and amplitude (Cc) in Krebs's solution containing 5 μM BMI and 1 μM strychnine (n = 13). Each point with error bar represents mean ± SEM. Numbers on the graphs (Cb) indicate the corresponding time of the traces sampled. Representative EPSCs evoked by two pulses (interval, 50 ms) of Vt stimulation (Da). During DHPG, the amplitude of the first EPSC was slightly decreased, whereas that of the second EPSC increased (*upper traces*), resulting in an increase of paired pulse ratio (PPR, the second/the first EPSC). In traces (Da), the EPSC before DHPG was normalized to the amplitude of the first EPSCs during DHPG or after washout of DHPG (*dotted line*, normalized EPSCs). Histograms summarized the amplitude of the first EPSC (Db) and the PPR (Dc) before, during and after DHPG. The mean amplitude of EPSC was significantly increased after washout of DHPG, (Db, *P < 0.05, n = 5), whereas the mean PPR was significantly increased during DHPG (Dc, *P < 0.05, n = 8).

sEPSCs were recorded in Vo neurons (Fig. 1A), voltage-clamped at -70 mV in the presence of 5 μM bicuculline methiodide (BMI) and 1-2 μM strychnine to block inhibitory synaptic responses (*see *Methods). The sEPSCs were predominantly mediated by AMPA receptors because most of them were disappeared by 10 μM 2,3-dioxo-6-nitro-1,2,3,4-tetrahydrobenzo [f]quinoxaline-7-sulfonamide (NBQX), an AMPA/kainate receptor blocker (Fig. 1B). Bath application of (*S*)-3,5-dihydroxyphenylglycine (DHPG; 10 μM, 5 min), a selective group I mGluR agonist, rapidly and significantly increased both the frequency (230.7 ± 42.9% of baseline) and the amplitude (124.6 ± 6.8% of baseline) of sEPSC (n = 13; Fig. 1C; P < 0.01 vs. baseline) at 2.5 min from the start of the application. The increased frequency of sEPSC was recovered to the baseline period (107.1 ± 41.9% of baseline at 13-14 min; Fig. 1Cb), whereas the increased amplitude was only partially recovered (114.3 ± 6.0% of baseline at 7-9 min; Fig. 1Cc), indicating that the DHPG-induced increase of sEPSC amplitude is long-lasting.

On the other hand, we tested the DHPG on EPSCs evoked by electrical stimulation of Vt to investigate if DHPG also affects evoked and synchronized glutamate release from primary afferent terminals. Apparently, in five out of eight Vo neurons, the DHPG (10 μM, 5 min) caused a small decrease of EPSC amplitude during bath application but an increase of that after washout (Fig. 1Da). The mean changes of evoked EPSCs were 87.1 ± 8.1% of baseline during DHPG (P > 0.05) and 124.2 ± 8.3% of baseline at 3 min after washout (P < 0.05; Fig. 1Db). When mean paired-pulse ratio (PPR), a change of which designates a presynaptic source of the modulation of synaptic transmission, was measured in all eight Vo neurons, it was significantly increased during DHPG application (0.97 ± 0.09; P < 0.05; Fig. 1Dc), compared to that before DHPG (0.79 ± 0.08). These results indicate that group I mGluR activation also regulates the synchronized glutamate release from primary afferent terminals.

### Mediation of group I mGluR subtypes in the DHPG-induced increases of sEPSC frequency and amplitude

**Figure 2:**
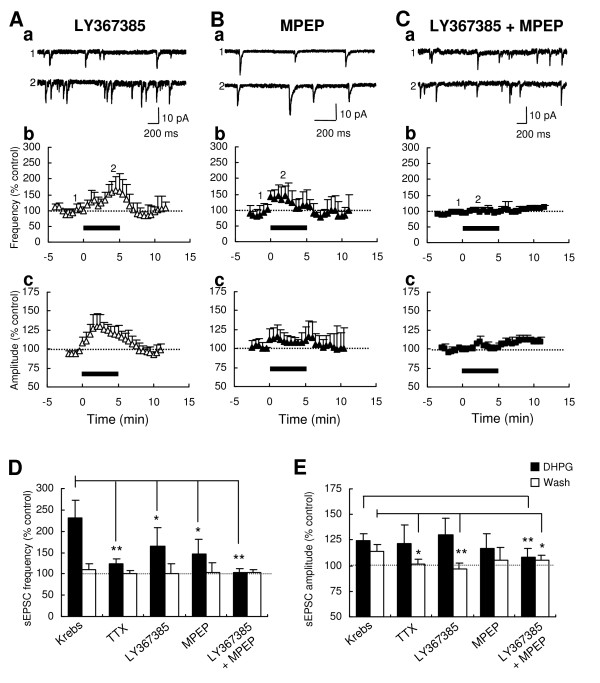
**Pharmacological characterization of the DHPG-induced increase of sEPSC frequency and amplitude**. Representative sEPSC traces, recorded from Vo neurons, before and during bath application of 10 μM DHPG (5 min) in the presence of the selective mGluR1 antagonist LY367385 (100 μM; Aa), the selective mGluR5 antagonist MPEP (10 μM; Ba), and LY367385+MPEP (Ca). Time-course graphs demonstrate the DHPG-induced changes of mean frequency (Ab, Bb and Cb) and amplitude (Ac, Bc and Cb) in LY367385 (n = 5), MPEP (n = 4) and LY367385+MPEP (n = 5). Numbers on the graphs indicate the corresponding time of the traces sampled. Histograms compare the magnitudes of DHPG-induced increases in sEPSC frequency (D) and amplitude (E) during the application (*filled*) or the washout (*open*) of DHPG in the presence of different antagonists including TTX (1 μM; n = 4), a Na^+ ^channel blocker. Asterisks indicate significant differences of DHPG effects, compared to the effect in the Krebs condition (**P < 0.01; *P < 0.05).

To identify which subtype(s) of group I mGluRs is responsible for the DHPG-induced increase of sEPSC frequency and amplitude, we tested DHPG in the presence of (+)-2-methyl-4-carboxyphenylglycine (LY367385), a selective mGluR1 antagonist, and/or 2-methyl-6-phenylethynyl-pyridine (MPEP), a selective mGluR5 antagonist. In the presence of LY367385 (100 μM), the DHPG-induced increase of sEPSC frequency was significantly reduced with a delayed peak at 4.5 min (164.7 ± 42.8% of baseline, n = 5; P < 0.05 vs. Krebs; Fig. 2Ab and 2D), but the increased amplitude was maintained (130.4 ± 15.9% of baseline; P > 0.05 vs. Krebs, Fig. 2Ac and 2E), compared to the Krebs condition. Both increases in frequency and amplitude were recovered to the baseline after washout of DHPG, indicating a requirement of mGluR1 for the long-lasting increase of sEPSC amplitude. Likewise, MPEP (10 μM) significantly reduced the DHPG-induced increase in the frequency, but not in the amplitude, of sEPSCs (frequency, 146.5 ± 33.9% of the baseline, n = 4, P < 0.05 vs. Krebs; Fig. 2Bb and 2D). Because individual LY367385 and MPEP partially or minimally inhibited the DHPG effects on the frequency and the amplitude of sEPSCs, we applied DHPG in the presence of both antagonists together. In this condition, the effects of DHPG on sEPSC frequency and amplitude were completely blocked (frequency, 102.9 ± 9.5% of baseline at 2-4 min, n = 5, P < 0.01; Fig. 2Cb and 2D; amplitude, 108.7 ± 8.2% of baseline at 2-4 min, P < 0.05; Fig. 2Cc and 2E). Taken together, these results indicate that mGluR1 and mGluR5 additively contribute to the glutamate release at presynaptic terminals in the Vo, while two subtypes compensate each other for the potentiation of postsynaptic responses.

Besides the identification of subtypes in the DHPG effects, we tested DHPG in the presence of 1 μM tetrodotoxin (TTX), a Na^+ ^channel blocker, to address what degree of action potential-independent glutamate release contributes to the DHPG-induced facilitation of glutamate release. In this condition (called miniature EPSC), DHPG-induced increase of sEPSC was significantly reduced in frequency (123.9 ± 10.2% of baseline at 2-4 min, n = 4; P < 0.01; Fig. 2D), but not in amplitude (Fig. 2E), compared to those in the Krebs condition, suggesting that the facilitating effect of DHPG on glutamate release was mainly due to an action potential-dependent mechanism. On the other hand, a recovery of the increased amplitude of sEPSC was observed at 7-9 min in the presence of TTX (102.0 ± 4.5% of baseline; Fig. 2E), presumably suggesting that a global neuronal excitability induced by the activation of group I mGluRs is required for the long-lasting increase of AMPA receptor-mediated synaptic responses.

### NMDA receptor-independent DHPG-induced increase of sEPSC frequency and amplitude

**Figure 3:**
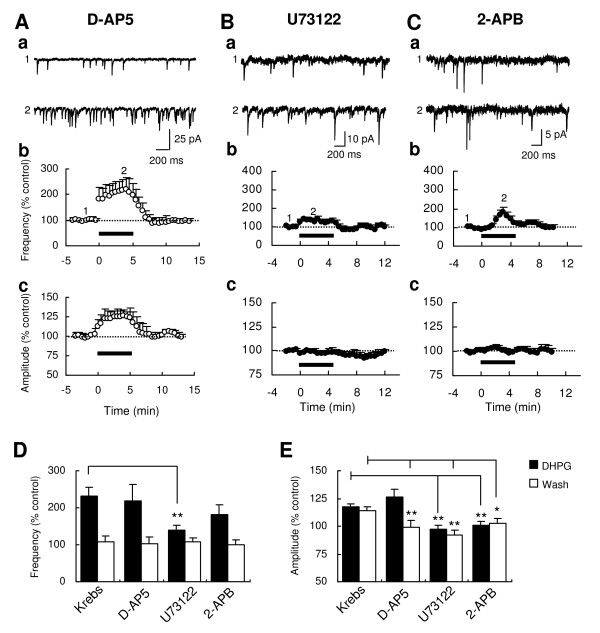
**Effects of D-AP5, U73122 and 2-APB on the DHPG-induced increase of sEPSC frequency and amplitude**. Representative sEPSC traces recorded from Vo neurons were sampled before and during bath application of 10 μM DHPG (5 min) in the presence of the NMDA receptor antagonist D-AP5 (50 μM; Aa), the PLC inhibitor U73122 (10 μM; Ba) and the IP_3 _receptor inhibitor 2-APB (100 μM; Ca). Time-course graphs demonstrate the DHPG-induced changes of mean frequency (Ab, Bb and Cb) and amplitude (Ac, Bc and Cb) in D-AP5 (n = 5), U73122 (n = 6) and 2-APB (n = 6). Numbers on the graphs indicate the corresponding time of the traces sampled. Histograms compare magnitudes of DHPG-induced increases in sEPSC frequency (D) and amplitude (E) during the application (*filled*) or the washout (*open*) of DHPG in the presence of D-AP5 (n = 5), U73122 (n = 6) and 2-APB (n = 6). Asterisks indicate significant differences of DHPG effects, compared to the effect in the Krebs condition (**P < 0.01, *P < 0.05).

To test if NMDA receptors mediate the DHPG-induced increases in the frequency and the amplitude of sEPSCs, DHPG was applied in the presence of 50 μM D-(-)-2-amino-5-phosphonopentanoic acid (D-AP5). In this condition, the increases of sEPSC frequency and amplitude by DHPG in the Krebs condition were not affected in the magnitude (frequency, 219.2 ± 45.2% of baseline, Fig. 3A and 3D; amplitude, 126.6 ± 7.0% of baseline; n = 5, Fig. 3E; both, P > 0.05 vs. Krebs). However, the long-lasting property of the increase of sEPSC amplitude by DHPG, shown in the Krebs condition was disappeared in the D-AP5 condition (98.9 ± 6.1% of baseline at 7-9 min; P < 0.01 vs. Krebs, Fig. 3Ac and 3E), indicating that NMDA receptors did not play a role in the DHPG-induced facilitation of glutamate release but in the long-lasting increase of AMPA receptor-mediated synaptic responses by DHPG.

### Involvement of phospholipase C pathway in the DHPG-induced increase of sEPSC frequency and amplitude

Group I mGluRs activate PLC via Gq/11, resulting in phosphoinositide hydrolysis, Ca^2+ ^release from IP_3_-sensitive intracellular stores and PKC activation [16]. Therefore, the involvements of PLC, IP_3 _receptor and PKC were tested. The blockade of the PLC with 1-[6-[[(17b)-3-methoxyestra-1,3,5(10)-trien-17-yl]amino]hexyl]-1H-pyrrole-2,5-dione (U73122, 10 μM) significantly reduced the DHPG-induced increases of sEPSCs in frequency (140.6 ± 12.6% of baseline at 2-4 min, n = 6, P < 0.01 vs. Krebs; Fig. 3B and 3D) and amplitude (97.6 ± 2.9% of baseline, n = 6, P < 0.01 vs. Krebs; Fig. 3E). In contrast, the blockade of the IP_3 _receptor with 2-aminoethoxydiphenylborane (2-APB, 100 μM) significantly inhibited the DHPG-induced increase of sEPSC not in frequency (181.9 ± 26.0% of baseline at 2-4 min, n = 6, P > 0.05 vs. Krebs; Fig. 3C and 3D), but in amplitude (101.0 ± 3.1% of baseline at 2-4 min, n = 6, P < 0.05 vs. Krebs; Fig. 3B and 3E), indicating a postsynaptic role of IP_3 _receptor.

**Figure 4:**
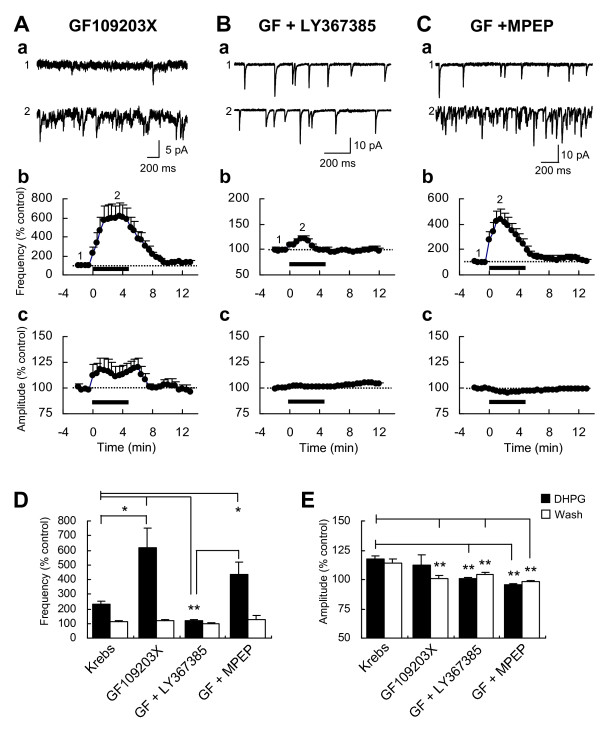
**Effects of GF109203X alone and with LY367385 or MPEP on the DHPG-induced increases of sEPSC frequency and amplitude**. Representative sEPSC traces before and during bath application of 10 μM DHPG (5 min) in the presence of the PKC inhibitor GF109203X (1 μM) alone (Aa) and with LY367385 (50 μM; Ba) or MPEP (10 μM; Ca). Time-course graphs demonstrate the DHPG-induced changes of mean frequency (Ab, Bb and Cb) and amplitude (Ac, Bc and Cb) in GF109203X (n = 7), GF109203X + LY367385 (n = 6) and GF109203X + MPEP (n = 5). Numbers on the graphs indicate the corresponding time of the traces sampled. Histograms compare magnitudes of DHPG-induced increases in sEPSC frequency (D) and amplitude (E) during the application (*filled*) or the washout (*open*) of DHPG in the presence of GF109203X (n = 7), GF109203X + LY367385 (n = 6) and GF109203X + MPEP (n = 5). Asterisks indicate significant differences of DHPG effects between two conditions indicated by lines (**P < 0.01; *P < 0.05).

On the other hand, blockade of PKC with 2-[1-(3-dimethylaminopropyl)indol-3-yl]-3-(indol-3-yl)maleimide (GF109203X, 1 μM) significantly enhanced the DHPG-induced increase of sEPSC in frequency (618.0 ± 135.1% of baseline at 2-4 min, n = 7, P = 0.01 vs. Krebs; Fig. 4A and 4D), but not in amplitude (111.9 ± 9.3% of baseline at 2-4 min, n = 7, P > 0.05 vs. Krebs; Fig. 4A and 4E). To further investigate which subtype of group I mGluRs is involved in the enhancement of the DHPG-induced increase of sEPSC frequency, we added either LY367385 or MPEP to the solution containing GF109203X. The addition of LY367385 to GF109203X prevented the enhancement of DHPG-induced increase of sEPSC frequency (120.6 ± 6.0% of baseline, n = 6; P < 0.01 vs. GF109203X or Krebs; Fig. 4B and 4D) and even more decreased the DHPG effect on the frequency, compared to that of LY367385 alone (Fig. 2A and 2D). However, the addition of MPEP to GF109203X caused only a small decrease of the enhancement by PKC inhibition (433.9 ± 85.2% of baseline, n = 5; P > 0.05 vs. GF109203X, P < 0.05 vs. Krebs; Fig. 4C and 4D). These results indicate that PKC plays an inhibitory role in the DHPG-induced increase of glutamate release, and mGluR1 is a main upstream activator of PKC at presynaptic terminals in Vo. On the other hand, the increased amplitude of sEPSC amplitude by DHPG was completely blocked when either LY367385 or MPEP was added to the GF109203X-containing solution (respectively, 101.3 ± 0.6% and 95.9 ± 0.6% of baseline; Fig. 4B, C and 4E). Together, these results suggest that two diverging signaling pathways from group I mGluR-activated PLC differently regulate presynaptic glutamate release and postsynaptic response at Vo synapses.

### Restricted roles of NO pathway in the DHPG-induced increase of sEPSC amplitude

**Figure 5:**
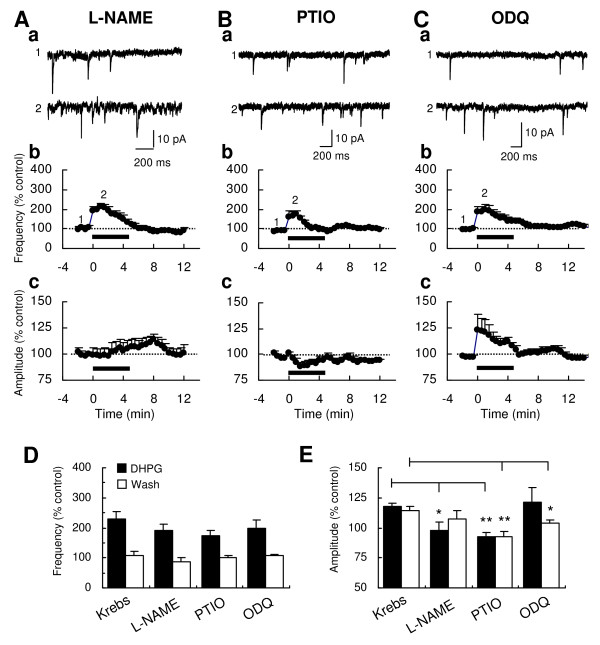
**Effects of L-NAME, PTIO and ODQ on the DHPG-induced increases of sEPSC frequency and amplitude**. Representative sEPSC traces before and during bath application of 10 μM DHPG (5 min) in the presence of the NOS inhibitor L-NAME (100 μM; Aa), the NO scavenger PTIO (10 μM; Ba) and the NO-sensitive guanylate cyclase inhibitor ODQ (10 μM; Ca). Time-course graphs demonstrate the DHPG-induced changes of mean frequency (Ab, Bb and Cb) and amplitude (Ac, Bc and Cb) in L-NAME (n = 5), PTIO (n = 6) and ODQ (n = 6). Numbers on the graphs indicate the corresponding time of the traces sampled. Histograms compare magnitudes of DHPG-induced increases in sEPSC frequency (D) and amplitude (E) during the application (*filled*) or the washout (*open*) of DHPG in the presence of L-NAME (n = 5), PTIO (n = 6) or ODQ (n = 6). Asterisks indicate significant differences of DHPG effects, compared to the effect in the Krebs condition (**P < 0.01; *P < 0.05).

Activation of mGluR1 augments cGMP accumulation [12], which is attributable to the sequential events of nitric oxide synthase (NOS) activation, NO production and NO-sensitive guanylate cyclase (GC) activation. It has been established that the activation of NOS is due to an increased intracellular Ca^2+ ^concentration from the Ca^2+ ^influx through Ca^2+ ^channels and/or Ca^2+ ^release from IP_3_-sensitive intracellular stores [22]. Therefore, an attempt was made to study the NO pathways in the DHPG-induced increases of sEPSC frequency and amplitude. We blocked the NOS with NG-nitro-L-arginine methyl ester hydrochloride (L-NAME, 100 μM), scavenged NO with 2-(4-carboxyphenyl)-4,4,5,5-tetramethylimidazoline-1-oxyl-3-oxide (PTIO, 10 μM), or blocked NO-sensitive NO-GC with 1H-[1,2,4]oxadiazolo [4,3-a]quinoxalin-1-one (ODQ, 10 μM). All three compounds did not significantly change the magnitude of DHPG-induced increase of sEPSC frequency at 2-4 min (L-NAME, 192.2 ± 19.9% of baseline, n = 5; PTIO, 175.5 ± 17.4% of baseline, n = 6; ODQ, 198.3 ± 27.1% of baseline, n = 6; P > 0.05 vs. Krebs; Fig. 5). However, these compounds had significant effects on the DHPG-induced increase of sEPSC amplitude during DHPG application (L-NAME, 97.5 ± 7.7% of baseline at 2-4 min, P < 0.05 vs. Krebs, Fig. 5A and 5E; PTIO, 92.6 ± 3.7% of baseline at 2-4 min, P < 0.01 vs. Krebs, Fig. 5B and 5E) or after washout of DHPG (ODQ, 103.5 ± 3.2% of baseline at 10 min, P < 0.05 vs. Krebs, Fig. 5C and 5E), indicating postsynaptic roles of the NO pathway in the action of group I mGluRs at Vo synapses.

### Involvements of CaMKII and ERK in the DHPG-induced increase of sEPSC frequency and/or amplitude

**Figure 6:**
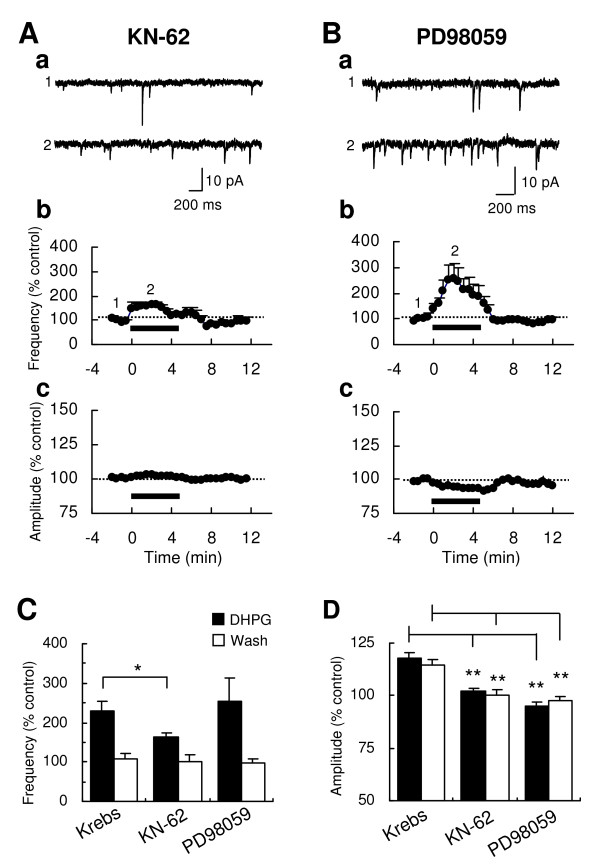
**Effects of KN-62 and PD98059 on the DHPG-induced increases of sEPSC frequency and amplitude**. Representative sEPSC traces before and during bath application of 10 μM DHPG (5 min) in the presence of the CaMKII inhibitor KN-62 (3.5 μM; Aa) and the MEK inhibitor PD98059 (50 μM; Ba). Time-course graphs demonstrate the DHPG-induced changes of mean frequency (Ab, Bb and Cb) and amplitude (Ac, Bc and Cb) in KN-62 (n = 5) and PD98059 (n = 6). Numbers on the graphs indicate the corresponding time of the traces sampled. Histograms compare magnitudes of DHPG-induced increases in sEPSC frequency (D) and amplitude (E) during the application (*filled*) or the washout (*open*) of DHPG in the presence of KN-62 (n = 5) or PD98059 (n = 6). Asterisks indicate significant differences of DHPG effects, compared to the effect in the Krebs condition (**P < 0.01; *P < 0.05).

The activation of group I mGluRs leads to the activation of many protein kinases [17], including extracellular signal-regulated kinase (ERK) in the spinal cord [17,23-26]. In addition, Ca^2+^-calmodulin-dependent protein kinase II (CaMKII) can be activated by elevated intracellular calcium as a result of release from intracellular stores and/or an influx through openings of various Ca^2+ ^channels [16]. Therefore, we tested the involvement of CaMKII and ERK in the DHPG-induced increases in the frequency and the amplitude of sEPSCs. Blockade of CaMKII with 4-[(2S)-2-[(5-isoquinolinylsulfonyl)methylamino]-3-oxo-3-(4-phenyl-1-piperazinyl)propyl] phenyl isoquinolinesulfonic acid ester (KN-62, 3.5 μM) significantly reduced the DHPG-induced increase of sEPSC in frequency (163.2 ± 11.9% of baseline at 2-4 min, n = 5, P < 0.05 vs. Krebs; Fig. 6A and 6C) and amplitude (102.3 ± 1.4% of baseline at 2-4 min, n = 5, P < 0.01 vs. Krebs; Fig. 6A and 6D). On the other hand, an inhibition of ERK activation by 2-(2-amino-3-methoxyphenly)-4H-1-benzopyran-4-one (PD98059, 50 μM), a mitogen-activated protein kinase kinase (MEK) inhibitor, showed a significant blockade of the DHPG effect on the amplitude (94.7 ± 2.1% of baseline at 2-4 min, n = 6, P < 0.01 vs. Krebs; Fig. 6B and 6D), but not on the frequency (253.4 ± 60.8% of baseline at 2-4 min, n = 6, P > 0.05 vs. Krebs; Fig. 6B and 6C), of sEPSCs. These results indicate that CaMKII and ERK are important protein kinases in the regulation of glutamate release and AMPA receptor-mediated synaptic responses by DHPG at Vo synapses.

### Expression of mGluR1 and mGluR5 in the Vo

**Figure 7:**
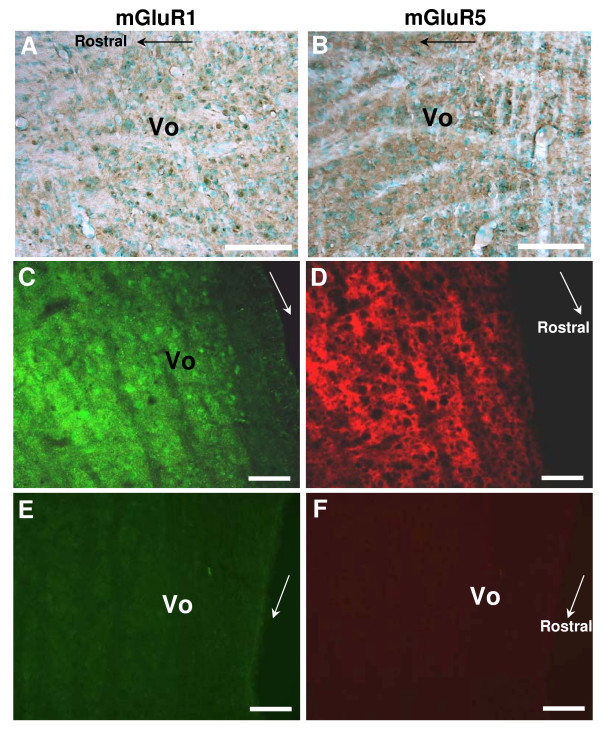
**Immunoreactivities of mGluR1 and 5 in Vo region**. Immunoperoxidase (A and B) and immunofluorescent (C and D) staining of mGluR1 (A and C) and mGluR5 (B and D) in Vo region of spinal trigeminal nucleus. Some arrow heads (A and C) designate cell body staining. The fluorescent images (C and D) are acquired from the same section with different filters. Scale bars equal 100 μm. Negative controls for the fluorescent images are shown (E and F). The arrows in images indicate the rostral direction.

Following the DHPG effects in Vo that are mediated by mGluR1 and/or 5, we attempted to identify the receptors in Vo with immunohistochemical/fluorescent staining methods. The immunohistochemical staining demonstrated distinct immunoreactive cells sporadically in Vo region for mGluR1 (Fig. 7A), but weak diffuse immunoreactivities for mGluR5 (Fig. 7B). Similarly, the immunofluorescent staining confirmed the distinct cell body-like immunoreactivities for mGluR1 (Fig. 7C) and the diffuse immunoreactivities for mGluR5 (Fig. 7D), indicating potential expressions of mGluR1 in cell bodies and mGluR5 at axon terminals in Vo.

## Discussion

The present study demonstrated that the bath application of DHPG markedly increased the frequency of sEPSCs recorded in Vo neurons, consequently indicating a facilitation of glutamate release by the activation of group I mGluRs from presynaptic terminals. Even though inhibitory effects of group I mGluRs on glutamate release have been widely discovered in the various brain regions [16], including the hippocampus [27] and the spinal cord [28], there have been few studies reporting the facilitating effects [16,29]. Therefore, the present study provides a new example of the facilitating effect of group I mGluRs on glutamate release in the CNS. Although the sources of glutamate were not clear since sEPSCs recorded from the Vo are mainly occurred by spontaneous unsynchronized glutamate release from terminals of other brainstem neurons, local Vo interneurons or trigeminal ganglion neurons, we found partial but significant mediation of mGluR1 or mGluR5 for the presynaptic facilitating effect of DHPG. In addition, the DHPG-induced facilitating effect was not dependent on the activation of NMDA receptors but voltage-dependent Na+ channels and CaMKII. The dependence of voltage-dependent Na+ channels indicates that an action potential-dependent mechanism for glutamate release is for the most part responsible for the DHPG-induced facilitating effect. Further, the interesting finding shown in the present study is that PLC and CaMKII mediate the facilitating effect of DHPG on the sEPSC frequency, whereas PKC negatively mediates it because its inhibition markedly enhances the facilitating effect of DHPG. The enhancement of DHPG effect caused by PKC inhibition involves mGluR1, rather than mGluR5. Our data together reveal that spontaneous glutamate release in Vo is strongly regulated by activation of group I mGluRs depending on activated signal transduction pathways.

AMPA receptors, iGluRs, consist of heteromeric assemblies of four different subunits GluR1-4, and mediate fast excitatory synaptic transmission at glutamatergic synapses in the CNS [30]. Diverse electrophysiological studies have indicated that the AMPA receptors are modulated by activation of group I mGluRs [16]. Group I mGluRs-mediated modulation of AMPA receptors includes both potentiation and depression, and is achieved by various intracellular signal transduction molecules downstream to group I mGluRs [12,31]. In this study, we demonstrated that DHPG-induced long-lasting potentiation of AMPA receptors-mediated sEPSC amplitude was completely blocked only when both mGluR1 and mGluR5 were blocked, indicating that a single subtype of group I mGluR is enough for the postsynaptic potentiating effect. These results has been demonstrated in the spinal dorsal horn neurons [29], and are comparable with earlier studies showing potentiaiton of AMPA, an agonist of AMPA receptors, -induced responses by group I mGluR activation [32-35]. Interestingly, albeit the potentiation during DHPG application was not dependent on NMDA receptors and PKC but on PLC, IP_3 _receptors, NOS, CaMKII and ERK, the long-lasting property of the potentiation was disappeared by the blockades of NMDA receptors, as well as individual antagonists used to block signal transduction pathways. This result indicates that the long-lasting potentiation requires a full set of signal transduction molecules recruited after the activation of group I mGluRs.

Although the synaptic localization of mGluR1 and mGluR5 has been known in the spinal cord dorsal horn [20,21], it has not been clearly demonstrated in the Vo. Compelling anatomical and electrophysiological studies have indicated the presence of mGluR1 and mGluR5 in the neuropil of all the trigeminal nuclei [15]. In the present study, the immunohistochemical data demonstrate cell body-like immunostaining of mGluR1 and diffuse staining of mGluR5 in the Vo area. In addition, the electrophysiological data put forward the expression of both subtypes on postsynaptic membrane and at presynaptic terminals in the Vo area, respectively, because of no significant blockade of sEPSC amplitude and significant but partial inhibition of the DHPG-induced increase of sEPSC frequency by either LY367385 or MPEP. On the other hand, a previous western blot analysis revealed the abundant expression of mGluR5 in the trigeminal ganglion but almost no expression of mGluR1, suggesting that the subtype of group I mGluR expressing at central terminals of trigeminal primary afferents in the Vo is exclusively mGluR5 [36]. Therefore, the DHPG-induced increase of sEPSC frequency, found in the present study, is probably originated from the activation of both mGluR1 and mGluR5 at terminals of other brainstem neurons or local Vo neurons, as well as the activation of mGluR5 at trigeminal afferent terminals in Vo [36]. This interpretation was also supported by the result that MPEP even more effectively inhibited the DHPG effect on sEPSC frequency (Fig. 2D). Accordingly, mGluR5 possibly mediates the regulatory effect of DHPG on the synchronized glutamate release from central trigeminal primary afferents, although the effect of MPEP on the DHPG-induced increase of PPR has not been tested in the present study.

Although other signaling molecules or channels, which were not studied here, may be involved in the DHPG effects [16], some signal transduction pathways relating to synaptic regulation by mGluR1 and mGluR5 are sequentially represented in the present study. Particularly, the present study demonstrates CaMKII as a positive regulator and PKC as a negative regulator in the presynaptic terminals of Vo region. Because PKC inhibition caused enhancement of the DHPG-induced facilitation of glutamate release, it could be postulated that the activation of PKC might render feedback inhibition to the group I mGluR activation by certain mechanisms, for instance, a desensitization [17,37-39] which terminates further activation of mGluR1 and/or 5 and thereby prevents the facilitation of glutamate release. In the postsynaptic dendritic spine, the activation of both mGluR1 and mGluR5 elevates intracellular Ca^2+ ^concentration that is due to the IP_3_-mediated intracellular Ca^2+ ^release [40], which may lead to the activation of the Ca^2+^-sensitive enzyme NOS [22] and CaMKII, via the binding with Ca^2+^-bound protein calmodulin, and then the positive modulation of AMPA receptor channels. The activation of NOS can activate NO-sensitive GC, converting GTP to cGMP [41]. The cGMP may play a role in the long-lasting increase of AMPA receptor-mediated synaptic responses (Fig. 5E). On the other hand, in the postsynaptic dendritic spine, ERK may be activated by Gβγ or Homer proteins [42], rather than a calcium-dependent manner, potentiating channel function of AMPA receptors responded to glutamate.

Previous studies have indicated significant roles of Vo brainstem area in the processing and the integration of somatosensory signals, including nociception [8,43-45]. It has been known that the Vo area contains convergent Vo neurons [46], the neurons also called wide-dynamic range neurons and responded to both non-noxious and noxious stimuli. The convergent Vo neurons are likely to be processors of somatosensory signals as well as mediators of wind-up phenomenon [47]. In addition to the convergent Vo neurons, neurons projecting their axons to the diencephalons have been found in Vo [9,10]. The diencephalic projections of Vo neurons were contralaterally or bilaterally reached to the various subnuclei of thalamus, such as ventral posteromedial nucleus and posterior thalamic nuclei, which involve the transmission of pain information into the higher brain areas. Therefore, the modulation of orofacial nociceptive signals in the Vo has functional significance in these contexts. In this study, we demonstrated the modulation of glutamate release and synaptic responses by the group I mGluR activation in the Vo. The modulation by group I mGluRs has been demonstrated in the central pain modulation area, such as the spinal cord dorsal horn. The activation of group I mGluR in the spinal cord dorsal horn induced long-lasting potentiation of the polysynaptic response [18] and, to the contrary, long-lasting depression of the monosynaptic response [18,19]. These studies *in vitro *have been correlated with other studies *in vivo *demonstrating group I mGluRs-mediated nociceptive sensitization [23,24,48,49]. Thus, our present finding, i.e., group I mGluRs-mediated augmentation of synaptic inputs into the Vo neurons, may support the notion that the amplification of somatosensory signals from the periphery by central pain transmission neurons underlies persistent pain [50].

In summary, we provided strong evidences that the activation of group I mGluR subtypes, mGluR1 and mGluR5, and their signal transduction pathways, differentially regulates glutamate release and AMPA receptor-mediated synaptic responses in the Vo region. These data will contribute to our understanding regarding the mode of the group I mGluR action adjusting brain functions such as orofacial normal sensation and pain.

## Materials and methods

Experiments were approved by Institutional Animal Care and Use Committee of Kyungpook National University, and were carried out in accordance with the National Institute of Health guidelines for the Care and Use of Laboratory Animals.

### Preparation of horizontal brain stem slices

Horizontal brainstem slices (400-450 μm) were prepared from 6-14 day-old Sprague-Dawley rats of either sex as described previously [51]. Under deep urethane anaesthesia (1.5 g/kg, i.p.), the brain and part of the cervical spinal cord were removed after decapitation, and then immediately transferred into an ice-cold Krebs' solution (composition in mM: NaCl 117, KCl 3.6, CaCl_2 _2.5, MgCl_2 _1.2, NaH_2_PO_4 _1.2, NaHCO_3 _25, and glucose 11; pre-oxygenated with a mixture of 95% O_2 _and 5% CO_2_; pH 7.4; CaCl_2 _was substituted with MgCl_2 _in case of the Ca^2+^-free medium). In the pre-oxygenated ice-cold Krebs' solution, other brain parts except for the brainstem were eliminated. The brainstem was glued upside down on the flat top of a hard mounting cube block that was prefixed to the bottom of a slice chamber. Then, horizontal slices were cut using a Vibratome 1000^+ ^(Vibratome, St. Louis, MO, USA). Typically, the first cut of the ventral part of the brainstem was discarded, and two horizontal brainstem slices were obtained. The slices prepared were transferred into a fresh oxygenated Krebs' solution at room temperature, and incubated at least an hour for an incubation that may wash hazardous molecules that occurred during the preparation processes. Either the right or the left side of the slice was moved to a recording chamber, and submerged and fixed with nylon strands drawn taut across a C-shaped sliver wire (~0.5 mm o.d.).

### Blind whole-cell voltage-clamp recordings

Blind whole-cell recordings with patch pipettes (borosilicate glass, TW150F; WPI, Sarasota, FL, USA) were made from Vo neurons. When viewed under a microscope (BX51WI, Olympus, Tokyo, Japan) with transmitted illumination (40×), the caudal border of the Vo area was distinguishable with the Vi area although it was difficult to discern with the rostral border of the Vo area [51,52]. Under visual guidance, the tip of the patch pipette was positioned in the Vo area above the caudal border with the Vi. The resistance of patch pipettes was typically 8-12 MΩ when filled with Cs-based (composition in mM: Cs_2_SO_4 _110, CaCl_2 _0.5, MgCl_2 _2, EGTA 5, HEPES 5, TEA chloride 5, ATP-Mg salt 5; Figs. 1, 2 and 3) or K-based (composition in mM: 145 K-gluconate, 5 NaCl, 1 MgCl_2_, 0.2 EGTA, 10 HEPES, 2 Mg-ATP, and 0.1 Na_3_-GTP; Figs. 3, 4, 5, 6 and 7) internal solutions (pH 7.2).

All recordings were made under a continuous perfusion of the preoxygenated Krebs' solution (2-3 ml/min), the same as the slice preparation solution, at room temperature (23-25°C). BMI (5 μM) and strychnine (1-2 μM) were always added to all the recordings to block inhibitory synaptic responses mediated by γ-aminobutyric acid type A (GABA_A_) and glycine receptors, respectively. After whole-cell formation was identified by appearance of the capacitance transients upon voltage pulses (-5 mV), sEPSCs were recorded at a holding potential of -70 mV. In this recording condition, sEPSCs were completely blocked by 10 μM NBQX or 6-cyano-7-nitroquinoxaline-2,3-dione (CNQX), AMPA/kainate receptor antagonists (Fig. 1B), indicating that the synaptic responses are mediated by the AMPA/kainate receptors. In addition, EPSCs were evoked in Vo neurons by two electrical stimuli (spaced at 50 ms) of Vt, and analyzed in their amplitudes and in PPRs. PPR was calculated by dividing the second EPSC by the first EPSC in amplitude. Recordings were amplified with a Multiclamp 700A amplifier (MDS Inc., Toronto, Canada), and sampled at 5-10 KHz and filtered at 1-2 KHz. Series resistances are occasionally monitored in the beginning, throughout and at the end of experiments. The recordings were terminated or discarded if series resistance (about 8-20 MΩ) changed by more than 20%. Data acquisition was performed using pClamp software (version 10; MDS Inc., Toronto, Canada). After a stable baseline recording for 5-10 min, DHPG (10 μM), known as a selective group I mGluR agonist, was bath-applied for 5 min. Antagonists were added to the perfusing and also the DHPG-containing Krebs' solutions. Frequencies and amplitudes of sEPSC before and after bath-application of DHPG were analyzed using a template-matching method (Clampfit of pClamp software) followed by cut-off filtering of amplitude threshold (typically 2 - 3 pA). The analyzed data were represented as mean ± SEM (%) of the baseline (before DHPG) over time (bin, 30 sec). Statistical comparisons were made using Student's *t*-test. Statistical significance, P < 0.05 or P < 0.01.

### Immunohistochemical staining

Similar aged rats used in whole-cell recordings were perfused with 4% paraformaldehyde in 0.1 M phosphate buffered saline (PBS; pH 7.4), and their brainstems were collected. The brainstems were post-fixed overnight at 4°C, and cryoprotected overnight in 30% sucrose solution (in 0.1 M PBS) at 4°C. Cryostat sections (40 μm) were made in a similar way as the horizontal brainstem slice preparation for whole-cell recordings (*see *above). For 3,3'-diaminobenzidine (DAB) development, sections were incubated overnight at 4°C with either goat polyclonal anti-mGluR1a/b (1:50; sc-47131, Santa Cruz Inc., Santa Cruz, CA, USA) or rabbit polyclonal anti-mGluR5 (1:50; 06-451, Upstate, Lake Placid, NY, USA) as primary antibodies, followed by anti-goat IgG for 1 hr or anti-rabbit IgG for 2 hrs, respectively. Nuclei were stained with methyl green. For fluorescent staining, sections were incubated with anti-goat IgG-FITC for 2 hrs and anti-rabbit IgG-Cy3 for 2 hrs after incubating at room temperature with both primary antibodies (1:200 for both). The primary antibodies were not incubated in case of negative controls. Nuclei were stained with 4',6'-diamidino-2-phenylindole (DAPI) (*data not shown*).

### Chemicals

Drugs were dissolved as a stock in distilled water or dimethylsulfoxide (DMSO), and diluted more than 1000 times to the final concentrations in the oxygenated Krebs' solution. Drugs and their sources were as follows: 2-APB, D-AP5, BMI, CNQX, DHPG, LY367385, MPEP, GF109203X, KN-62, L-NAME, PD98059, PTIO, ODQ, strychnine and U73122 from Tocris Cookson (Ellisville, MO, USA); TTX from Sigma (St. Louis, MO, USA).

## Competing interests

The authors declare that they have no competing interests.

## Authors' contributions

All electrophysiological recordings were performed by JHS and SMH. JHS and ESP analyzed the electrophysiological records. ESP, SRH and DKA performed immunohistochemical experiments and analysis, or helped interpreting data. DHY designed the experiments, participated in data analysis and interpretation, and wrote this manuscript. All authors read and approved the final manuscript.
